# Assessment of near-infrared fluorophores to study the biodistribution and tumor targeting of an IL13 receptor α2 antibody by fluorescence molecular tomography

**DOI:** 10.18632/oncotarget.19569

**Published:** 2017-07-26

**Authors:** Parul Gupta, Jo-Ann Wentland, Mauricio Leal, Dangshe Ma, Rachel Roach, Antonio Esparza, Lindsay King, Mary E. Spilker, Cedo Bagi, Christopher T. Winkelmann, Anand Giddabasappa

**Affiliations:** ^1^ Global Science and Technology, Comparative Medicine, Pfizer, Inc., La Jolla, CA, USA; ^2^ Pharmacokinetics and Drug Metabolism, Pfizer, Inc., New York NY, USA; ^3^ Oncology Research Unit, Pfizer, Inc., Pearl River, NY, USA; ^4^ Center for Therapeutic Innovation, Pfizer, Inc., La Jolla, CA, USA; ^5^ Comparative Medicine, Pfizer, Inc., La Jolla, CA, USA; ^6^ Current affiliation: Regeneron Pharmaceuticals, Tarrytown, NY, USA

**Keywords:** biotherapeutics, tumor targeting, biodistribution, fluorescence molecular tomography (FMT), molecular imaging

## Abstract

Non-invasive imaging using radiolabels is a common technique used to study the biodistribution of biologics. Due to the limited shelf-life of radiolabels and the requirements of specialized labs, non-invasive optical imaging is an attractive alternative for preclinical studies. Previously, we demonstrated the utility of fluorescence molecular tomography (FMT) an optical imaging modality in evaluating the biodistribution of antibody-drug conjugates. As FMT is a relatively new technology, few fluorophores have been validated for *in vivo* imaging. The goal of this study was to characterize and determine the utility of near-infrared (NIR) fluorophores for biodistribution studies using interleukin-13 receptor subunit alpha-2 antibody (IL13Rα2-Ab). Eight fluorophores (*ex/em*: 630/800 nm) with an N-hydroxysuccinimide (NHS) linker were evaluated for Ab conjugation. The resulting antibody-fluorophore (Ab-F) conjugates were evaluated *in vitro* for degree of conjugation, stability and target-binding, followed by *in vivo*/*ex vivo* FMT imaging to determine biodistribution in a xenograft model. The Ab-F conjugates (except Ab-DyLight800) showed good *in vitro* stability and antigen binding. All Ab-F conjugates (except for Ab-BOD630) resulted in a quantifiable signal *in vivo* and had similar biodistribution profiles, with peak tumor accumulation between 6 and 24 h post-injection. *In vivo*/*ex vivo* FMT imaging showed 17–34% ID/g Ab uptake by the tumor at 96 h. Overall, this is the first study to characterize the biodistribution of an Ab using eight NIR fluorophores. Our results show that 3-dimensional optical imaging is a valuable technology to understand biodistribution and targeting, but a careful selection of the fluorophore for each Ab is warranted.

## INTRODUCTION

Antibody-based therapeutics have revolutionized cancer treatment due to their impressive target-specific activity. Biodistribution studies during preclinical biotherapeutic drug development are cornerstones for elucidating their target specificity, efficacy, and potential on- or off-target toxicity [1]. Traditional biodistribution and pharmacokinetic studies can utilize a number of experimental approaches, including dynamic nuclear 3D imaging techniques such as positron emission tomography (PET) and single photon emission computed tomography (SPECT), invasive sampling at various time points followed by a radioactivity measurement, a ligand-binding assay (LBA) and/or immunohistochemistry [2–4]. PET and SPECT imaging can be used as non-invasive methods for biodistribution studies but require specialized skill sets and highly regulated labs for radioisotope handling and may be limited by the short half-life of some radionuclides. Fluorescence-based *in vivo* imaging techniques can overcome some of these limitations. Our lab is assessing the utility of fluorescence molecular tomography (FMT) in evaluating the biodistribution of biotherapeutics. For example, we recently determined the *in vivo* biodistribution and tumor uptake of a 5T4 antibody-drug conjugate (ADC) and the 5T4 antibody itself using FMT [5]. FMT is an advanced optical imaging technology that uses the near-infrared spectrum (NIR) (600–900 nm) for non-invasive *in vivo* imaging and 3D quantification of the fluorescent probes [5–7]. As compared to lower wavelength probes, NIR probes have the advantages of deeper signal penetration in tissue and minimal background signal because tissue biomolecules do not significantly absorb light in the NIR range [6, 8–11]. Additionally, the spectral resolution of FMT allows the simultaneous detection of multiple fluorophores without an additional step of spectral unmixing. FMT can provide non-invasive and quantitative data in mouse models, similar to radionuclide-based imaging. There are a variety of NIR fluorophores currently available for conjugation to primary amines on proteins that can be used in FMT imaging [12, 13]. However, there is limited literature on the utility of these fluorophores for *in vivo* biodistribution studies using optical imaging technologies.

The objective of this study was to evaluate and determine the utility of different NIR fluorophores (commercially available) for antibody conjugation and biodistribution studies in mice. An antibody against the interleukin-13 receptor subunit alpha-2 (IL13Rα2), an antigen found to be over-expressed in many cancer types was used as the model compound [14–17]. Eight different amine-reactive NIR fluorophores (Table 1, λ_max_: 630–800), BODIPY-X630/650^®^, VivoTag^®^645, Alexa Fluor^®^647, VivoTag680^®^, AlexaFluor680^®^, AlexaFluor750^®^, IRDye800CW^®^ and DyLight800, were conjugated to the IL13Rα2-Ab. These antibody-fluorophore (Ab-F) conjugates were evaluated *in vitro* for conjugation efficiency, stability and binding to the antigen, while *in vivo* biodistribution in a mouse tumor xenograft model was evaluated using FMT imaging. The results from this study compare the biodistribution of an antibody when conjugated to different NIR fluorophores.

**Table 1:** Comparison of fluorophore and Ab-F properties

Fluorophore	Molecular Weight Daltons	ε (Fluorophore) cm^−1^ M^−1^	λEx max nm	Degree of Labeling (DOL)	Injected Dose (mg/kg)
BODIPY630	660.5	101000	625	1.3	5.8
VT645	1393	210000	643	2.3	3.3
AF647	1250	239000	650	2.5	3.0
VT680	1856	210000	668	2.4	3.1
AF680	1150	184000	679	3.3	2.3
AF750	1300	290000	752	2.3	3.3
IR800Dye	1166	240000	778	1.4	5
DyLight800	1050	270000	770	2.5	N/A

For each fluorophore, the molecular weight (Da), emission coefficients (ε: cm^−1^ M^−1^) and absorption maxima (*ex/em*: nm) are provided. The degree of labeling obtained for each Ab-F and respective *in vivo* doses (mg/kg) that equate to a 2 nmol dose of fluorophore are shown in the last two columns.

## RESULTS

### *In vitro* evaluation of Ab-F conjugates

#### SEC-HPLC profiling

SEC-HPLC was used to determine if fluorophore conjugation caused any changes in the stability (aggregation) of the Ab. The elution profiles of the Ab before and after fluorophore conjugation were compared in terms of the retention time (RT) of the major peak (abundance > 90%). Molecules of larger size elute first and smaller molecules elute later. The RT of the Ab before fluorophore conjugation was 15.7 min and the RT of all Ab-F conjugate samples were between 15.4–15.8 min after conjugation, except for DyLight800 (Supplementary Figure 1 and Table 2). The RT corresponding to the major peak in the DyLight800 conjugate was 19.5 min, whereas the peak with the RT of 15.8 min (corresponding to the major peak in other samples) comprised only 7% of the total peak area. The significant shift in RT and relatively small peak area under the 15.8 min peak for the DyLight800 sample suggest significant changes in the stability of this Ab-F conjugate [18]. All other Ab-F conjugates had similar RTs as the unconjugated Ab.

**Table 2:** SEC-HPLC analysis of Ab-F conjugate

Label	RT (min)	%Area
Unlabeled Ab	15.7	95.9
BOD630	15.4	89.0
VT645	15.5	95.3
AF647	15.6	95.8
VT680	15.5	95.8
AF680	15.7	87.3
AF750	15.6	95.3
IRDye800	15.3	89.8
DyLight800	19.5	46.9

The stability of Ab-F conjugates was evaluated using SEC-HPLC analysis. The retention time (RT) and the Area (%) of Ab-F conjugates was compared to the RT of the unconjugated Ab (15.7 min). All Ab-F conjugates showed an RT between 15.3–15.7, except DyLight800, whose major peak had an RT of 19.5 min.

### Cell binding assay for the Ab-F

**Figure 1:**
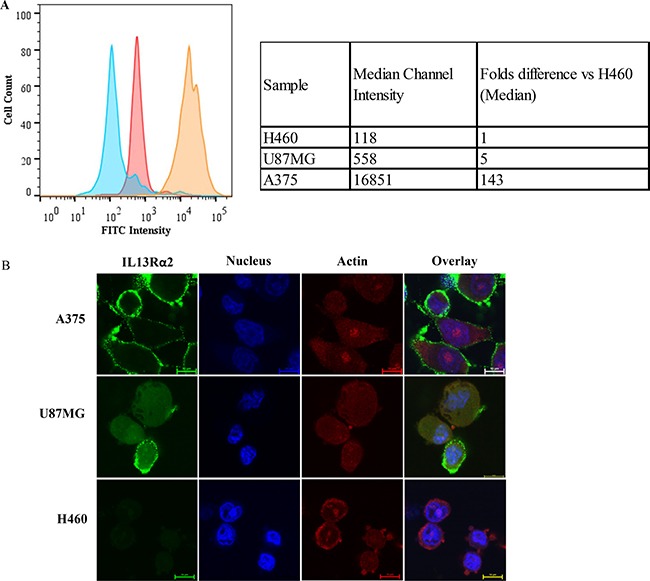
IL13Rα2 expression in three cell lines The relative expression of IL13Rα2 was evaluated in A375, U87MG and H460 cell lines by (**A**) flow cytometry and (**B**) fluorescence microscopy. (A) The histogram shows the FITC fluorescence intensity for each sample plotted against cell count (Histogram color: Blue - H460; Pink – U87MG; Orange – A375). The table (inset) shows a quantitative comparison of the median channel intensity for each cell line and also relative expression of A375 and U87MG, compared to H460 cells. (B) Representative fluorescence microscopy images (20× magnification) of A375, U87MG and H460 cells stained for IL13Rα2 (FITC: green), actin (AlexaFluor594: red) and nucleus (DAPI: blue). All experiments were performed in triplicates and repeated two times.

The cell lines A375, U87MG and H460 were tested for IL13R α2 expression using flow cytometry and immunofluorescence microscopy. The flow cytometry results showed that A375 cells have the highest expression of IL13Rα2, followed by U87MG cells, whereas H460 cells express minimal levels of IL13Rα2 (Figure 1A). Compared with that in H460 cells, the binding of IL13Rα2 Ab was 5- and 143-folds higher in U87MG and A375 cells, respectively (Figure 1A). These observations were also confirmed by immunofluorescence microscopy. Formalin-fixed cells were incubated with the IL13Rα2 primary Ab, which was detected using a FITC-conjugated secondary Ab. Microscopy images showed that the IL13Rα2 was primarily localized on the cell membrane and that A375 cells had the highest expression of IL13Rα2 (+++) followed by U87MG cells (+), while H460 (−) cells had minimal expression (Figure 1B).

**Figure 2:**
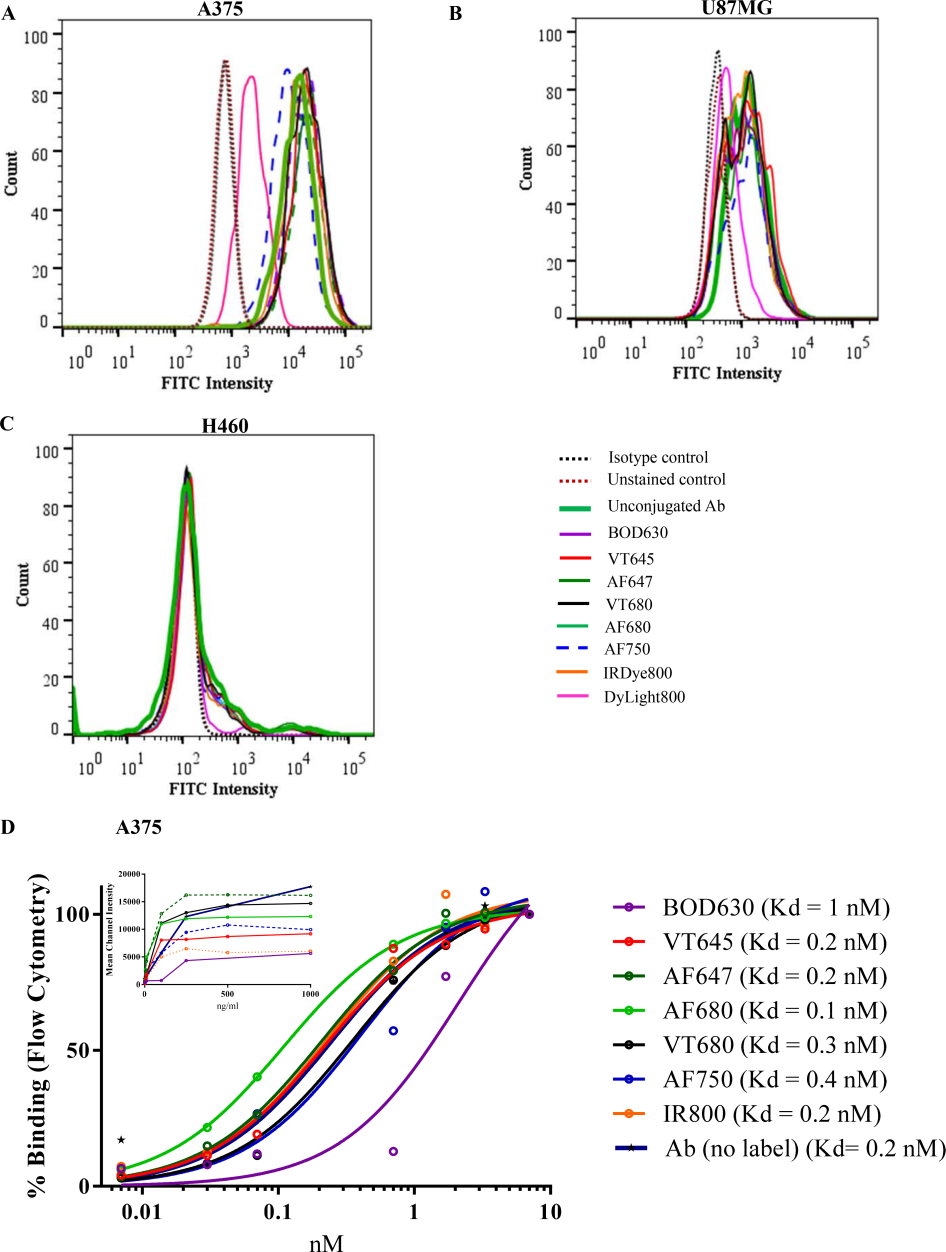
Cell binding assay The cell binding ability of Ab-F conjugates was assessed by flow cytometry in (**A**) A375, (**B**) U87MG, (**C**) H460 cell lines. The cells were incubated with different Ab-F conjugates or unlabeled Ab, followed by detection with a FITC-conjugated secondary Ab. Cells incubated with an isotype control Ab were used as the negative control (Isotype control), cells incubated with unlabeled Ab were used as the reference standard (unconjugated Ab) and cells that were not labeled with any Ab were used as the unstained control. The peak shifts after labeling with detection Ab (FITC-conjugated secondary antibody) was compared among treatments. The histogram for the DyLight800-conjugated Ab showed a peak shift from that of the unconjugated Ab in A375 and U87MG cells. All other Ab-F conjugates showed a peak shift similar to the unconjugated Ab and thus the histograms overlap on each other. (**D**) Receptor binding of IL13Rα2 Ab conjugated to different fluorophores to A375 cells. All Ab-F conjugates showed highest binding at ∼100–200 ng/mL Ab concentration. All experiments were performed in triplicates and repeated two times.

Flow cytometric analysis was performed to evaluate the effect of fluorophore conjugation on the ability of IL13Rα2 Ab to bind to cell surface antigen. The cells (A375, U87MG and H460) were incubated with Ab or Ab-F, followed by detection with a FITC-labeled secondary Ab. The flow cytometry results showed that conjugation of the Ab with NIR fluorophores did not change its cell-binding properties, except in the case of DyLight800 (Figure 2A–2C). With the exception of DyLight800, all other Ab-F conjugates had average mean channel fluorescence intensity (MFI) within 115% of the unlabeled Ab in all three cell lines. The MFI for the DyLight800-conjugated Ab was 15% and 37% of the unlabeled Ab in A375 and U87MG cells, respectively. The H460 cells showed negligible binding with the Ab as well the Ab-F. These results suggest that DyLight800 conjugation reduced the cell-binding of Ab significantly and hence this conjugate was excluded from further studies. Additionally, concentration-dependent cell binding was evaluated with selected Ab-F conjugates (Ab-BOD630, -VT645, -AF647, -AF680, -VT680, AF-750 and -IR800) and unlabeled Ab in A375 cells by flow cytometry (Figure 2D). The results showed that all Ab-F conjugates (except –Ab-BOD630) had similar concentration-dependent cell binding profiles, although the observed maximum intensity varied among different fluorophores. The saturation of binding for all Ab-F conjugates was observed at an Ab concentration of 100–200 ng/mL (Figure 2D, inset). Additionally the concentrations of Ab-F required for half-maximal binding (analogous to Kd; relative binding potency for various Ab-F) were about 0.2 ± 0.1 nM for all the groups except Ab-BOD630 which was 2.0 nM (Figure 2D).

### Evaluation of the biodistribution of IL13Rα2 Ab-F conjugates by FMT imaging

#### *In vivo* analysis

**Figure 3:**
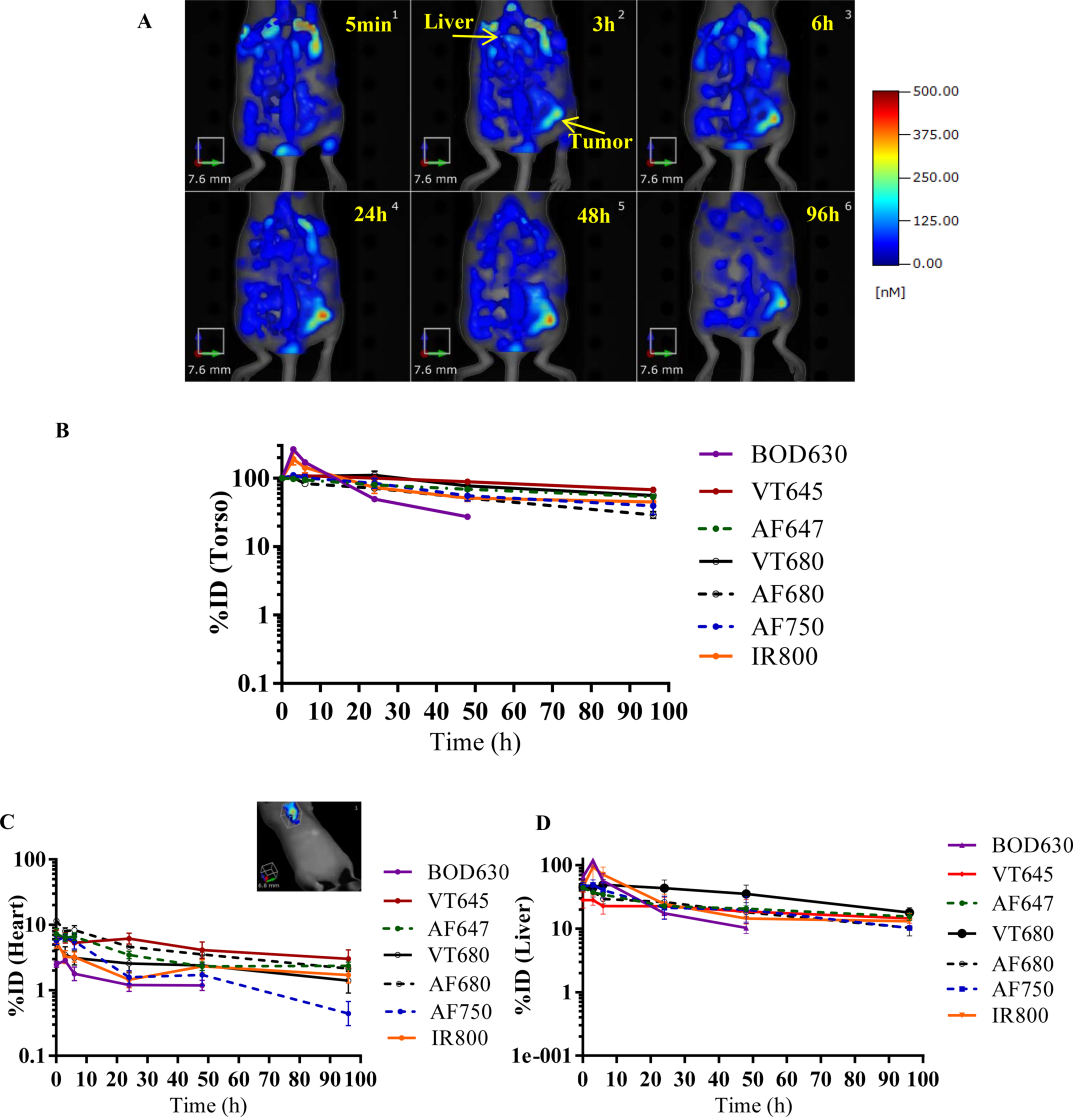
*In vivo* FMT imaging of the whole-body, heart and liver Non-invasive longitudinal imaging was performed on the whole-body and the heart at 5 min and 3, 6, 24, 48 and 96 h after injection of Ab-F conjugates in A375 xenograft bearing mice. (**A**) Representative FMT images of a mouse at different time points. The image shows whole-body (torso) fluorescence in a mouse from the Ab-AF680 group. (**B**) The whole-body and (**C**) heart FMT profile (%ID vs. time) of various Ab-F conjugates. The inset in ‘C’ shows the heart ROI. (**D**) liver FMT profile (%ID vs. time) of various Ab-F conjugates. The profiles are averages from *n* = 5/group, with ± SEM as error bars.

The Ab-F conjugates that passed the *in vitro* quality control assessment were evaluated for biodistribution and tumor targeting in the mouse A375 xenograft model. The A375 cell line was selected because it had the highest expression of the target antigen, IL13Rα2. The Ab-F conjugates (Ab-BOD630, -VT645, -AF647, -VT680, -AF680, -AF750, and -IRDye800) were injected intravenously into xenograft-bearing mice, followed by longitudinal FMT imaging (5 min to 96 h). Representative time-course images of mice injected with Ab-AF680 are shown in Figure 3A. These FMT images show high fluorescence signal throughout the whole-body at 5 min post-injection, followed by a sustained reduction in whole-body fluorescence signal, except in the tumor where the signal increased over time. Quantitation of whole-body images (whole-body and torso have been used interchangeably) shows a similar decrease in signals after 5 min for most of the Ab-F conjugates, with the exception of the Ab-BOD630 and -IR800 conjugates, which show a spike in the whole-body signal at 6 h, followed by a continuous decline (Figure 3B). Quantitation of the heart signal was evaluated as a surrogate for plasma clearance (Figure 3C). Interestingly, the heart profiles of Ab-BOD630, -AF750 and -IR800 conjugates showed faster clearance compared to other fluorophore conjugates. Additionally, significant difference was observed in whole-body and heart at later time point which may be attributed to differential metabolism of fluorophores. ANOVA analysis of the area under the curve and Cmax for the torso and heart (obtained from time vs. %ID curves) from different groups showed that the values for BOD630 and IR800 were significantly different from those for all other groups (*p* < 0.05). *In vivo* FMT liver profiles were similar for Ab-VT645, -AF647, -AF680 and -AF750 conjugates (Figure 3D). Ab-BOD630 and -IR800 showed a spike similar to the whole-body profile for these conjugates at 6 h time point. Interestingly, Ab-VT680 showed significantly higher liver accumulation at all time-points compared to other fluorophore conjugates. This may be due to preferential metabolism and accumulation of Ab-VT680 in the liver (Figure 3D).

**Figure 4:**
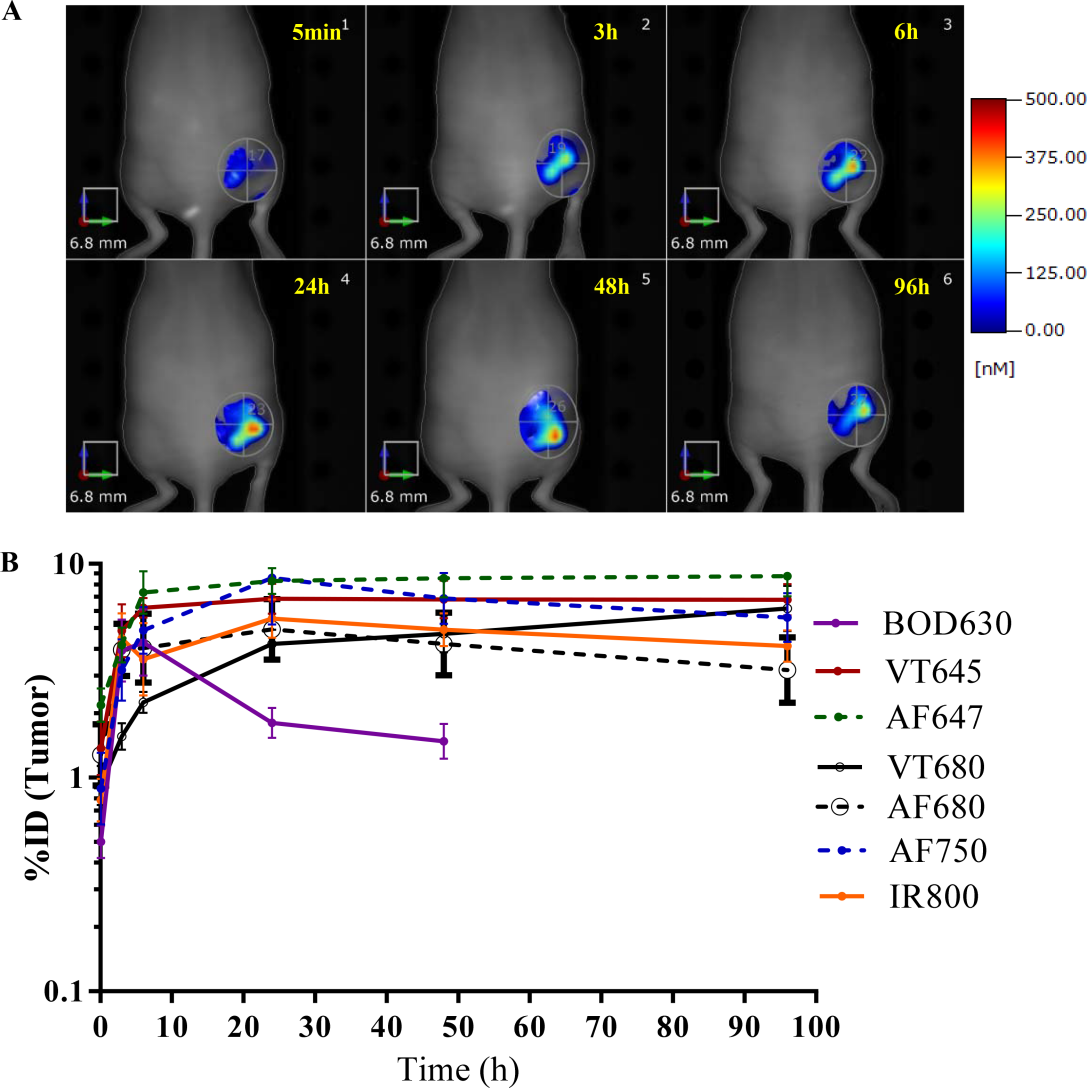
*In vivo* FMT imaging of the tumor Non-invasive longitudinal imaging was performed on the whole-body at 5 min and 3, 6, 24, 48 and 96 h after injection of Ab-F conjugates in A375 xenograft bearing mice. An ROI was created around tumor to quantitate Ab uptake in tumors. (**A**) Representative tumor ROI images of a mouse from the Ab-AF680 group at various time points. (**B**) FMT quantitation for Ab uptake in the tumor (%ID) vs. time for various Ab-F conjugates. The profiles are averages from *n* = 5/group, with ± SEM as error bars.

Figure 4A is a representative time-course panel showing tumor accumulation of Ab-AF680 in mice. As expected, due to the tumor-targeting properties of the IL13Rα2 Ab, the images show a continuous increase in Ab-AF680 signal until 48 h post-injection (Figure 4A). The tumor concentrations for all Ab-F conjugates (except Ab-BOD630) peaked between 6–24 h post-injection and were maintained until 96 h after dosing (Figure 4B). Unlike other groups, the tumor signal in the Ab-BOD630 group decreased drastically (more than a 50% reduction) after the 6 h time point (Figure 4B) and was not detectable *in vivo* at 96 h. Table 3 shows the tumor uptake of Ab expressed as % ID/g calculated from the *in vivo* and *ex vivo* FMT images at 96 h. The mean tumor volume and weights at 96 h for between all, but Ab-AF647 group were similar (Supplementary Figure 2). Statistical analysis of the *in vivo* tumor uptake at 96 h for all Ab-F compounds (excluding –Ab-BOD630) showed a mean tumor uptake of 23.8 ± 2.2% ID/g with a 95% confidence interval of 19.3–28.3% ID/g. An ANOVA on the area under the curve for tumor profiles showed that uptake of Ab-BOD630 was significantly less (*p* < 0.05) than the other compounds, but the Cmax for tumors was not found to be significantly different for any group (Figure 4B). Overall *in vivo* FMT imaging data showed effective and sustained tumor uptake of the Ab across all the Ab-F groups (except for the Ab-BOD630 group).

**Table 3:** Comparison of *in vivo* and *ex vivo* Ab uptake by tumor at 96 h in different Ab-F groups

Ab-F	*In vivo* (% ID/g ± SEM)	*Ex vivo* (% ID/g ± SEM)
BODIPY630	n.d.	5 ± 1
VT645	34 ± 6	31 ± 3
AF647	24 ± 2	23 ± 3
VT680	26 ± 5	15 ± 3
AF680	18 ± 5	12 ± 3
AF750	24 ± 6	24 ± 5
IR800Dye	17 ± 5	19 ± 5

The Ab uptake was calculated from the *in vivo* and *ex vivo* images as %ID/g using the torso (whole-body) FMT signal at 5 min as the total dose.

### *Ex vivo* FMT analysis

**Figure 5:**
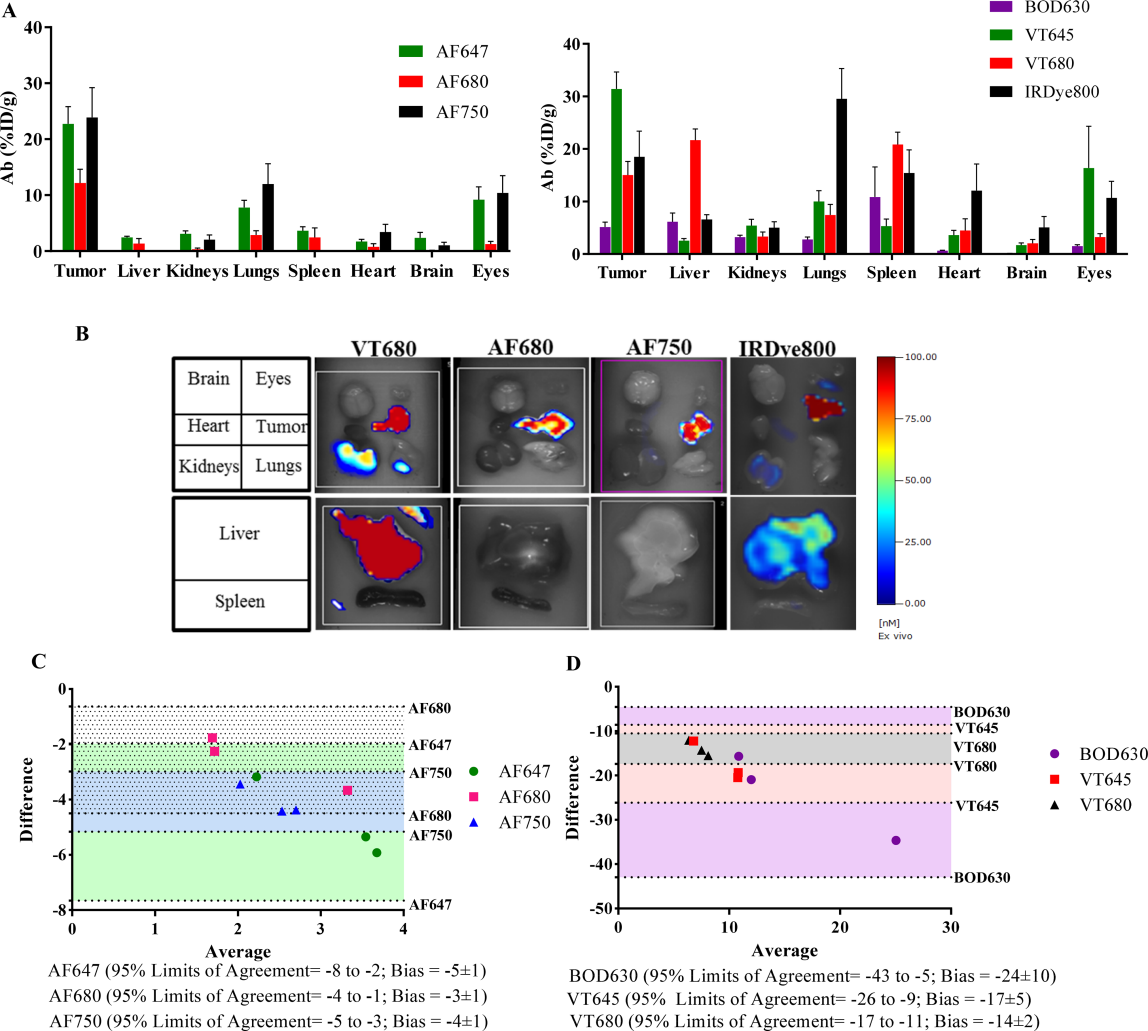
Comparison of *ex vivo* FMT imaging and LBA quantitation *Ex vivo* FMT imaging was performed at 96 h on selected organs collected after whole-body perfusion. (**A**) Ab uptake in terms of %ID/g for organs from various Ab-F groups. (**B**) Representative images for organs (brain, eyes, heart, tumor, kidneys, lungs, liver and spleen) from the Ab-VT680, Ab-AF680, Ab-AF750 and Ab-IRDye800 groups. (**C**) And (**D**) Bland-Altman plot for comparison of quantitative values obtained from FMT and LBA for the tumor. The Ab-F conjugated to AF647, AF680 and AF750 showed narrow limits of agreement (95%) and lower bias (C), whereas Ab-F conjugated to BOD630, VT645 and VT680 showed wider limits of agreement (95%) and higher bias. *n* = 3–4/group, with ± SEM as error bars.

*Ex vivo* 3D FMT imaging and quantitation was performed on selected tissues - tumor, liver, spleen, heart, kidneys, lungs, brain and eye at 96 h, after whole-body perfusion of the mice to eliminate signal interference from blood remaining in the vasculature. Figure 5A shows the organ % ID/g from each Ab-F group at 96 h, whereas representative *ex vivo* FMT images from selected Ab-F (-VT680, -AF680, -AF750 and -IRDye800) are shown in Figure 5B. The overall tumor uptake was observed to be 12–31% ID/g for all the groups, except the Ab-BOD630 group, which showed only 5% ID/g tumor uptake for the Ab (Figure 5A and Table 3). Hence, the Ab uptake in the tumors as well as in the other organs for the Ab-BOD630 group showed the highest deviation from the corresponding values of all other Ab-F groups. The mean tumor Ab uptake estimated by all Ab-F groups (except -BOD630) was 20.6 ± 1.8% ID/g with a 95% confidence interval of 16.9–24.4% ID/g. Notably, the mean tumor uptake from *ex vivo* imaging was about 87% of the tumor uptake calculated from *in vivo* imaging, suggesting an approximately 13% blood/vascular contribution to the total Ab uptake by tumors evaluated by *in vivo* imaging at 96 h. However, we observed high variability for Ab uptake in non-tumor tissues relative to the uptake in tumor tissues for the different fluorophores (Figure 5A), which may be reflective of differential metabolism, retention and elimination of these fluorophores. The AF647, AF680, AF750 and VT645 groups showed at least 3-fold higher accumulation in the tumor than in any other organs, while the BOD630, VT680 and IR800 groups showed relatively high signal in all the tissues with some tissues showing Ab uptake comparable to that in the respective tumors (i.e., organs such as the lungs, liver, heart or spleen; Figure 5A, 5B and Supplementary Table 1).

### Ab quantitation in tissue and blood samples by LBA

Ligand binding assay (LBA) is a routine method for determining the concentration of an Ab in plasma and tissues. Hence, LBA was used to determine the amount of IL13Rα2 Ab in the tumor, for all the groups (Table 4). LBA detects the intact Ab-F only, where as FMT detects the fluorophore from intact and also metabolized Ab-F fragments. At 96 h, the Ab concentrations determined by LBA in the tumors from all the groups was 0.4–1 μg/g, except for the Ab-BOD630 (4 μg/g) and Ab-IRDye800 (which was below the detection limit) groups, and the Ab concentration in tumors from the unlabeled Ab group was 2 μg/g (Table 4). To compare with the LBA results, the concentrations calculated from FMT image analysis were converted to μg/g using their respective average DOL values. In contrast to LBA quantitation, FMT data showed significantly higher tumor Ab-F concentrations for all the groups tested. After conversion, the Ab concentrations in the tumors calculated from FMT from the AF647, AF680, AF750 and IRDye800 groups were 3–6 μg/g; the concentrations for the VT645 and VT680 groups were 15–20 μg/g; and for the Ab-BOD630 group was 29 μg/g (Table 4). The higher concentrations in the Ab-BOD630 group may be attributed to the higher dose of Ab-BOD630 due to the decreased DOL. We further used a Bland-Altman plot for statistical comparison of the tumor Ab concentrations obtained from LBA versus those obtained from FMT. The analysis showed that the AF647, AF680 and AF750 groups had the narrowest 95% limits of agreement: -8 to -2, -4 to -1 and -5 to -3, respectively (Figure 5C). The same groups also showed lower bias values of -5, -3 and -4, respectively. On the other hand, Ab-VT645, VT680 and BOD630 showed wider 95% limits of agreement with much higher bias values (Figure 5D). The narrow 95% confidence limits of agreement and lower bias values from the Bland-Altman analysis suggest a better statistical correlation of LBA with FMT data for the AlexaFluor^®^ dyes than for the VivoTags^®^ and BODIPY^®^. In addition, pharmacokinetic (PK) profile in blood samples was evaluated for the unlabeled-Ab and Ab conjugated to AF680 and AF750. The PK time profile and the quantitative parameters for Ab conjugated to AF680 or AF750 were similar to the unlabeled Ab (Supplementary Figure 3).

**Table 4:** Comparison of Ab uptake by *ex vivo* FMT imaging and LBA

Ab-F	FMT (μg/g)	LBA (μg/g)
**BOD630**	29 ± 4	4 ± 2
**VT645**	20 ± 2	0.8 ± 0.2
**AF647**	6 ± 1	0.7 ± 0.1
**VT680**	15 ± 2	0.4 ± 0.0
**AF680**	3 ± 0.6	1 ± 0.3
**AF750**	4 ± 2	0.4 ± 0.1
**IRDye800**	4 ± 1	BLQ*
**Unlabeled Ab**	NA	2.2 ± 1.8
* Below Limit of Quantitation		

The *ex vivo* FMT Ab uptake data were converted into units of μg/g for the comparison. The table shows the *ex vivo* Ab quantitation in μg/g obtained by LBA and FMT for the tumors from different Ab-F groups.

## DISCUSSION

Antibodies and other biotherapeutics are uniquely suited for labeling and detection by fluorescent probes [19]. These proteins typically have lysine residues with free amines that can be conjugated to amine-reactive fluorophores using NHS (N-hydroxysuccimide) chemistry [20]. Previously, our lab and others have shown that VT680XL, an amine-reactive NIR fluorophore, can be conjugated to antibodies or ADCs to study organ distribution and estimate pharmacokinetic parameters by non-invasive FMT imaging [5, 6, 21]. Similar examples use different fluorophore-conjugated Abs for biodistribution studies with the IVIS Lumina Imaging System (Perkin Elmer) [6, 22–25]. In contrast to IVIS, FMT imaging is specialized for 3D imaging of deep tissues and volumetric quantitation of fluorophores with picomolar sensitivity, *in vivo* and *ex vivo* [5, 6]. Three dimensional optical imaging will give higher resolution and isotropic data, similar to PET/SPECT imaging and will help in better understanding tissue and tumor biodistribution. Currently, there is no data to compare and evaluate the performance of different fluorophores for the rational design of imaging-based biodistribution studies of antibodies. This study is the first to evaluate multiple amine-reactive NIR fluorophores for Ab conjugation and to characterize their performance by non-invasive 3D optical imaging.

Fluorophores such as IRDye800^®^CW, VT680^®^, XenoLight CF680 and XenoLight CF750 have been used to label proteins (albeit different proteins) via NHS chemistry and the protein quality can be ensured by thin layer chromatography, HPLC or immune-binding assays [6, 23–27]. The DOL which represents the number of fluorophore molecules conjugated to each molecule of Ab is a critical parameter that should be balanced for good *in vivo* signal, at the same time retaining the properties of Ab. Labeling of an Ab molecule with more than 4 fluorophore molecules (DOL > 4) can have deleterious effects on protein binding, functional activity and biodistribution [27, 28]. Thus, we optimized the labeling method by varying the protein:fluorophore ratios to achieve an average DOL of 1–3, to enable enough sensitivity for extended periods by FMT imaging at a relevant biological dose. Our results showed that a DOL of 1–3 formed stable Ab-F conjugates and did not affect the binding to the target antigen (IL13Rα2), except in the case of the DyLight800 conjugate. An earlier study with 5T4-ADC conjugation to VT680 (DOL of 3) also showed no significant difference in cellular binding activity compared to the parent Ab [5]. Although DyLight800 had similar spectral properties and molecular weight as the other fluorophores, it had a significant effect on IL13Rα2-Ab stability and cellular binding. However, in a separate study, when DyLight800 was conjugated to another Ab (for a target other than IL13Rα2), no significant change in cell binding was observed, suggesting that the impact of Ab conjugation can be specific to each Ab (data not shown). Hence, the conjugation of DyLight800 to the IL13Rα2 Ab might affect the steric interactions between Ab-F conjugate molecules, overall molecule charge or other biochemical properties specific to this particular Ab-F combination [29–31]. This suggests that each Ab-F conjugation is unique, and assays to qualify them for *in vivo* evaluation should be performed. For other Ab-F conjugates tested in this study, a DOL of 1-3 provided a quantifiable FMT signal without affecting the stability or *in vitro* binding to the antigen, thus facilitating the *in vivo* evaluation.

*In vivo* FMT imaging data provided biodistribution profiles for the IL13Rα2 Ab-F conjugates in the whole-body, the liver, the tumor and the heart. Co-registration with computer tomography (CT) would probably allow for *in vivo* quantitation of additional organs. Heart profiles were used as a surrogate for blood concentrations and showed faster clearance for Ab conjugated to AF750 than for Ab conjugated to VT645, AF647, VT680 or AF680. Evaluation of the FMT data from tumors for all fluorophore groups showed that the peak concentrations of Ab occurred between 6–24 h post-injection. At 96 h significant variations in concentration of Ab-F conjugates was observed in whole-body, heart and tumor. Such inter-fluorophore differences are probably due to the differential residualization of the fluorophores in the tissues, alternative pathways for metabolism and elimination of these probes [25]. A better understanding of the fluorophore metabolism and clearance will help in understanding the free fluorophore contribution. Alternatively, the difference at 96 h may be due to the technical issues like FMT sensitivity or the limitations of tomographic reconstruction for a fluorophore at very low levels.

There are limited data available comparing the biodistribution of an antibody using different platforms and probes. For example, Oliveira *et. al*. and Aerts *et al*. compared EGFR mAb uptake using two imaging platforms, PET (^89^Zr) and fluorescent imaging (IRdye800) [25, 32]. Oliviera *et al*. showed that, for a majority of organs, the %ID/g values for EGFR Ab (dually conjugated to IRDye800 and ^89^Zr) calculated using NIR fluorescence were similar to the values obtained from gamma ray quantification (using ^89^Zr) [25]. However, Ab concentrations for the tumor and kidney were higher when calculated by NIR signal than when calculated from the ^89^Zr signal, while the NIR-calculated liver concentration was lower than the ^89^Zr measurement. In another study anti-mesothelin mAb uptake was compared after labeling with ^89^Zr and IR-800 [33]. Although the trends for tumor uptake were similar between 2D-optical and microPET imaging, the 2D-optical imaging did not allow for longitudinal *in vivo* quantitative comparison of whole-body profile and tumor uptake. Such limitations of resolution and quantitation can be addressed by using a 3D-tomographic optical imaging modality.

Our results also showed overall higher Ab concentrations in the tumor as estimated by FMT than the concentrations estimated by LBA. These differences may be due to the different species detected by FMT and LBA in calculating the Ab concentrations. LBA measures intact anti-IL13Rα2 Ab and not its metabolites and thus reflects the concentration of Ab at the time of measurement. LBA may also sometimes be confounded by cross reactive or soluble forms of the antigen. Anti-IL13Rα2 Ab used in this study does not cross react with the mouse isoform (data not shown) and human cells do not shed or have soluble isoforms of this receptor [34]. Thus interference from soluble isoform of mouse or human tumor cells is not expected in this LBA assay. FMT is an indirect method that measures Ab concentration based on signal from the fluorophore, which may include full length Ab as well as its metabolized Ab fragments and thus we anticipated higher values than LBA. In addition, Ab-F might be processed by dissimilar metabolic and elimination pathways depending on the fluorophore attached resulting in quantitative differences [27].

The fate of radiolabels or fluorophores is influenced by conjugation chemistry and biophysical properties, which can alter their permeability, residualization and to a certain extent the charge of the probe [31, 35–37]. The charge of the fluorophore can also affect the properties of the Ab like antigen binding, Fc receptor interaction, recycling, rate of diffusion across tissues and thus causing changes in biodistribution and PK of the Ab [30]. Similar to radiolabels, the charge on fluorophores can influence their catabolism and excretion or retention by cells as degraded protein adducts [31]. Labels that are catabolized and eliminated at a faster rate without residualization may yield accurate quantities of intact Ab at any given time. However, labels that residualize within cellular compartments after catabolism tend to accumulate over time and represent a cumulative quantity rather than reflecting the actual temporal concentration [31, 35–37]. Thus, some fluorophores may have similar characteristics to those of residualizing radiolabels. A comparison of three different classes of dyes, cyanine based dyes, boran-based dyes (BODIPY) and smaller polycyclic dyes (oxazine- or thioazine-based), revealed no correlation between their residualizing properties and their optical or physicochemical properties [38]. However, AF680, AF750, IRDye800 and BOD650 were retained significantly within the cell, albeit at different rates [38]. Alternatively, some fluorophores may exhibit increased fluorescence when the Ab-F is degraded due to unquenching of the signal after loss of conjugation, contributing to variations in the relative quantitations from different fluorophores [38]. In this study we found a spike in *in vivo* fluorescence for BOD630 and IR800 at 6 h post injection. Additionally, we observed significantly higher tumor concentrations by FMT in VT680, VT645 and BOD630 groups (Table 4). Such differences may be attributed to the higher residualization of these fluorophores or increase in fluorescence due to un-quenching of signal [39]. We also observed significantly higher concentrations in liver and spleen for -VT680; and spleen, lungs and heart for –IR800 (Supplementary Table 1). This may be due to preferential metabolism and residualization of these fluorophores or fluorophore conjugates in these organs.

Overall, this study provides a comprehensive comparison and cross-validation of eight different amine-reactive NIR fluorophores, with *ex/em* wavelengths ranging from 630–800 nm, for conjugation of an antibody and measurement of its biodistribution in a tumor-bearing mouse xenograft model. This is the first study to discuss the behavior of multiple fluorophores conjugated to the same Ab and thus improves the current understanding of Ab-F conjugates and their utility in biodistribution studies. We show that conjugation of IL13Rα2 Ab with most of the NIR fluorophores evaluated did not affect binding and stability of the Ab. It would be ideal to perform such a quality control assessment case by case while studying a different Ab. We think 3D optical imaging (like FMT) is a good tool to understand the profile of whole-body, tissue distribution and targeting. FMT imaging provided a qualitative and a semi-quantitative assessment of the Ab accumulation in potential target tissues and tumor. In this study with IL13Rα2 Ab, we found that AlexaFluor class of fluorophores showed lower overall FMT signal, but similar profiles to each other and better correlation with LBA. VivoTag fluorophores and BOD630 were less correlative to LBA and also the VivoTag class of fluorophores showed higher overall signal in all tissues. Based on this observation, we think if specificity is an important parameter AlexaFluor may be the choice fluorophore, whereas VivoTag may be useful in detecting very low levels of signal or target antigen. In summary, this study provides a foundation for the wider use of antibody-fluorophore conjugates in biodistribution studies, tumor targeting, biomarker and PK/PD studies in preclinical research. Advanced mathematical models to interpret and calculate PK parameters from imaging data has shown good utility [40, 41]. Adapting such kinetic modeling tools can further enhance the understanding of FMT imaging data. In the future, it will be interesting to perform a cross-platform (Optical, PET and SPECT imaging) comparison of biodistribution and targeting of IL13Rα2 Ab.

## MATERIALS AND METHODS

### Ethics statement

All animal related procedures performed in this study were in accordance with established guidelines and regulations, and were reviewed and approved by the Pfizer Institutional Animal Care and Use Committee. Animals were housed under standard 12:12 light:dark cycle in ventilated racks at a room temp of 72 F and relative humidity between 30% and 70%.

### Reagents

The IL13Rα2 Ab was generated by Pfizer Inc. The fluorophores VivoTag680XL^®^ (VT680) (#NEV11119) and VivoTag645^®^ (VT645) (#NEV11173) were obtained from Perkin Elmer. AlexaFluor647^®^ (AF647) (#A-20006), AlexaFluor680^®^ (AF680) (#A-20008), AlexaFluor750^®^ (AF750) (#A-20011), BODIPY-X630/650^®^ (BOD630) (#D-10000), DyLight800^®^ (#46421), rhodamine-conjugated phalloidin (#R415) and Prolong antifade mounting media (#P36962) were obtained from Life Technologies, and IRDye^®^800 (IRDye800) (#929-70020) was obtained from Licor. The Nunc^™^ Lab-Tek^™^ II Chamber Slide^™^ System (#12-565-8), DAPI and an anti-human fluorescein-conjugated secondary Ab against human IgG (#PI31529) were obtained from Thermo Fisher Scientific Inc. BIOT sheep anti-human IgG polyclonal Ab (#AU003.MCUS01) and AlexaFluor647^®^ goat anti-human IgG (#A80-319A) were obtained from Binding Site and Bethyl Labs, respectively.

### Cell culture

The A375, U87MG and H460 cell lines were obtained from the ATCC and were cultured according to the ATCC's instructions. Briefly, A375, U87MG and H460 cells were cultured in DMEM (#2017-01) (Gibco Life Technologies), EMEM (#12-125Q) (Lonza) and RPMI medium (#2021-05) (Gibco Life Technologies), respectively, supplemented with 10% fetal bovine serum (#SH30088) (GE Healthcare Life Sciences) in a 37°C incubator with 5% CO_2_. All experiments were performed before the cell lines reached passage 15.

### Ab-labeling with fluorophores

The fluorophores evaluated in this study have succinimidyl ester groups that react with the amine groups on proteins to form a stable amide linkage. The reactions were carried out in amine-free phosphate buffered saline (PBS) (Gibco Life Technologies). The Ab was incubated for 2 h in the dark with varying amounts of fluorophore in the labeling buffer. The conjugates were purified using Zeba^TM^ Spin Desalting Columns (7K molecular weight cut off) (#89891) (Thermo Fisher Scientific, Inc.) and the average degree of labeling (DOL) was calculated using a spectrophotometer (Nanodrop^TM^ 1000, Thermo Fisher Scientific, Inc.), where the DOL is defined as the average number of fluorophore molecules conjugated to each molecule of protein. The ratio of the amount of fluorophore to protein was optimized by multiple trials to achieve a DOL of 1 to 3 (Table 1).

### SEC-HPLC analysis

The aggregation, stability or purity of Abs was determined using size-exclusion chromatography high performance liquid chromatography (SEC-HPLC) after conjugation to the fluorophores. The reagents were stored at 4°C for a month before characterizing by SEC-HPLC. SEC-HPLC was performed using a YMC-Pack Diol-200 column (Waters Corporation) under isocratic conditions. The mobile phase consisted of 20 mM sodium phosphate and 400 mM sodium chloride. The proteins were detected by UV absorbance at 280 nm to determine the retention times of the major peak (the peak with the highest percentage area under curve).

### Cell binding flow cytometry

Cells were plated in six-well plates and incubated overnight. The following day, Ab or Ab-F conjugates were added to the cells at a concentration of 500 ng/mL. After 4 h of incubation at 37°C, the cells were washed and further incubated with a fluorescein-conjugated anti-human secondary Ab for 1 h at 37°C. The cells were washed and fixed with 10% formalin, followed by re-suspension in PBS and analysis by a MACS Quant Analyzer (Miltenyi Biotec). The median channel intensities for FITC were used to compare the cell binding properties of the Ab. All experiments were performed in triplicates and repeated two times.

### Immunofluorescence microscopy

A375, U87MG and H460 cells were plated overnight on coverslips. The following day, cells were washed, fixed with 10% formalin for 30 minutes and blocked with 5% BSA for 30 min. The cells were incubated with the primary IL13Rα2 Ab (500 ng/mL) overnight at 4°C, washed and then incubated with a secondary Ab conjugated to fluorescein (anti-human) (1:500). In addition, cellular β-actin was also stained using rhodamine-conjugated phalloidin for 1 h. Following incubation, the cells were washed and mounted using ProLong Diamond reagent. Images were acquired at 60× magnification using a Nikon T*i* microscope (Nikon Instruments, Inc.) using the appropriate filters for each fluorophore. All experiments were performed in triplicates and repeated two times.

### *In vivo* studies and FMT imaging

All *in vivo* studies were approved by the Pfizer Institutional Animal Care and Use Committee (IACUC). Six-eight week old NU-*Fox1^nu^* female athymic nude mice weighing about 25 g was purchased from Charles River Labs. To minimize the interference of mice diet on FMT imaging, they were kept under irradiated special diet AIN-76A PD (Newco Distributors Inc.). To generate xenograft tumors, approximately 2 × 10^6^ A375 cells in 50% Matrigel (#3432-005-01) (BME Pathclear, Trevigen) were implanted subcutaneously in the flanks of Fox1^nu^ female athymic nude mice, 6–8 week old and weighing about 25 g. The tumor volumes were calculated by measuring the longest perpendicular x and y axis of the tumors using Vernier calipers and using the formula (length × breadth^2^)/2 [42]. When the tumors reached a size of 200–500 mm^3^, the mice (*n* = 5/group) were administered the Ab-F conjugates intravenously via the tail vein at a dose equivalent to 2 nmol of the fluorophore (Table 1). While mice were anesthetized with isoflurane, whole-body imaging was performed using the FMT4000^®^ (Perkin Elmer) at 5 min and 3, 6, 24, 48 and 96 h after the administration of the Ab-F conjugates. The mice were euthanized at 96 h by whole-body perfusion with heparinized saline through the heart, and organs (tumor, brain, eyes, heart, lungs, liver, kidneys and spleen) were collected for *ex vivo* FMT imaging. After imaging, the liver, tumor and kidneys were weighed and flash-frozen using liquid nitrogen for quantitation of Ab using a ligand binding assay (LBA). The FMT imaging data were analyzed and quantitated using TrueQuant^TM^ software, as described previously [5, 6]. ROIs (regions of interest) around the torso and heart were used as surrogates for whole-body and blood clearance profiles, respectively. The 5 min torso signal was used as the total injected dose (100 %ID). The profiles are averages from *n* = 5/group, with ± SEM as error bars.

### Ligand binding assay for blood and tissue samples

The IL13Rα2 Ab concentrations in tissues (tumor, liver and kidneys) and blood were quantitated using the Gyrolab™ workstation-based sandwich immunoassay and fluorescence detection. The samples were prepared by diluting the whole blood or tissue homogenates or calibration standards in 3% bovine serum albumin and 0.1% Tween-20 (3% BSA PBST). A biotinylated sheep anti-human IgG antibody (100 μg/mL) was used as the capture protein for the tested Ab. The capture protein was loaded on streptavidin coupled micro-columns located on the Bioaffy^™^ CD (compact disc) within the workstation, followed by washing and sample loading. The samples were detected by a goat anti-human IgG AlexaFluor647^®^ antibody (5 μg/mL) using fluorescent laser detection, and the data were analyzed using the Watson v7.4 LIMS system (Thermo Fisher Scientific, Inc.) to determine the Ab concentrations (μg/g). The tumors collected from mice 96 h post-injection of the unlabeled Ab were used to compare the Ab uptake in tissues. A minimum of 3 samples were analyzed by LBA from each group.

### Statistical analysis

Statistical analysis was performed using Prism 6.0 (GraphPad Software, Inc.). The results are presented as mean ± S.E.M with *n* = 3–6/group. The data were analyzed using column statistics (Bland-Altmann plot) to evaluate the agreement between two modes of measurements [43]. The Bland-Altmann plot is a statistical tool to quantify agreement between two methods of quantitative measurements by constructing “limits of agreement” that are calculated from the mean and the standard deviation(s) of the difference between measurements. Differences were considered statistically significant when *p* < 0.05.

## SUPPLEMENTARY MATERIALS FIGURES AND TABLE



## Abbreviations

Ab: Antibody
Ab-F: Antibody-fluorophore conjugate
ADC: Antibody-drug conjugate
AF: AlexaFluor^®^
BOD: BODIPY-X^®^
DOL: Degree of labeling
FMT: Fluorescence molecular tomography
IL13R α2: IL13 receptor α2
LBA: Ligand-binding assay
NHS: N-hydroxy succinimidyl ester
NIR: Near-infrared
VT: VivoTag^®^
