# Supplementary material for: Assessment of near-infrared fluorophores to study the biodistribution and tumor targeting of an IL13 receptor α2 antibody by fluorescence molecular tomography
Source: Oncotarget. 2017 Jul 26;8(34):57231–45. doi: 10.18632/oncotarget.19569 (PMC5593638; doi:10.18632/oncotarget.19569)
Supplement: Supplementary file 1 [file oncotarget-08-57231-s001.pdf]

Assessment of near-infrared fluorophores to study the biodistribution and tumor targeting of an IL13 receptor α2 antibody by fluorescence molecular tomography

SUPPLEMENTARY MATERIALS

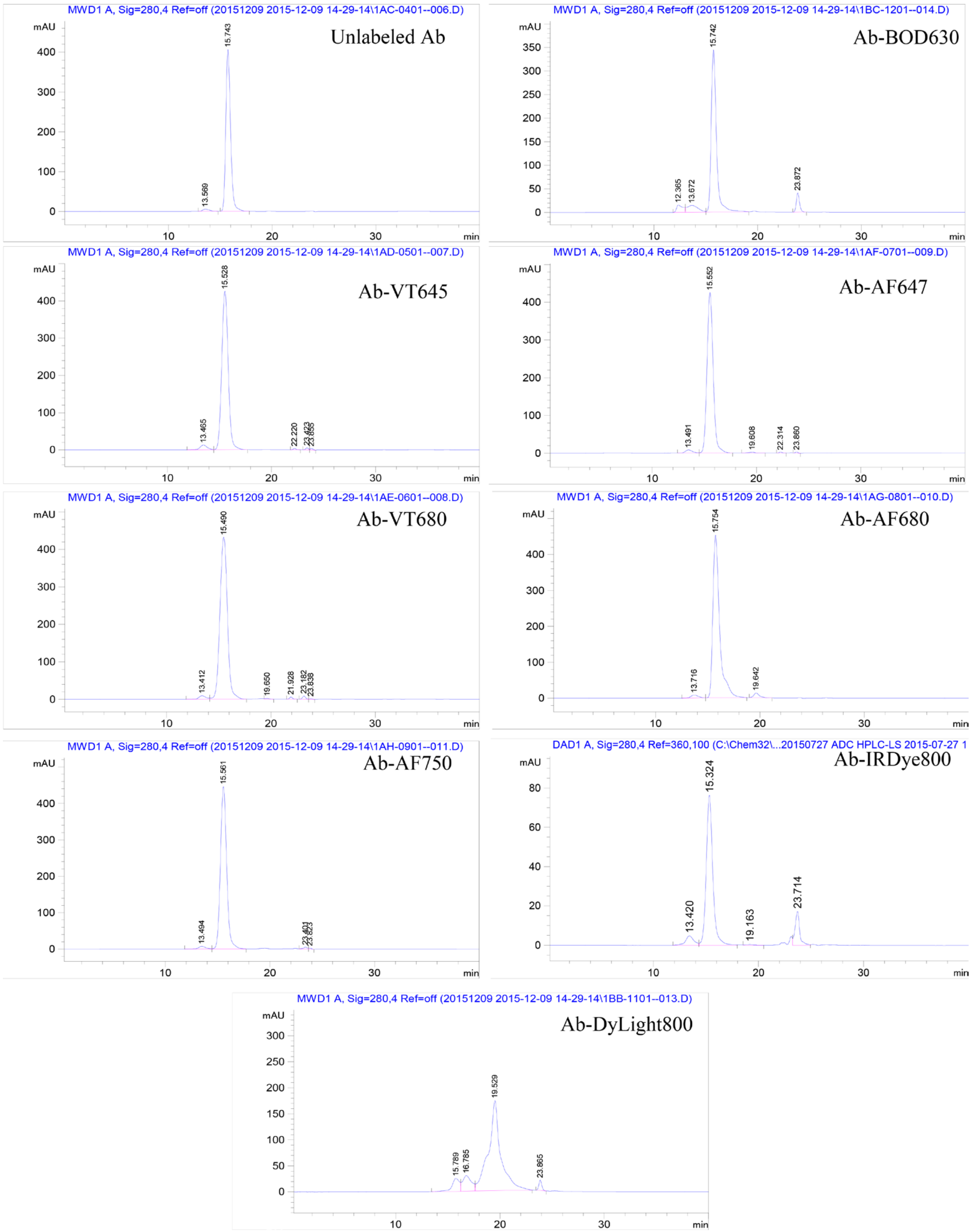

Supplementary Figure 1: SEC-HPLC chromatograms for each Ab-F conjugate. The figure shows SEC-HPLC chromatograms for the unlabeled Ab and each of the Ab-F conjugates with absorption units (mAU) on the y-axis and retention time (minutes) on the x-axis. The RT of the major peak was used to assess the stability and purity of the Ab-F conjugates. All the Ab-Fs, except Ab-DyLight800 showed similar profiles for the major peak.

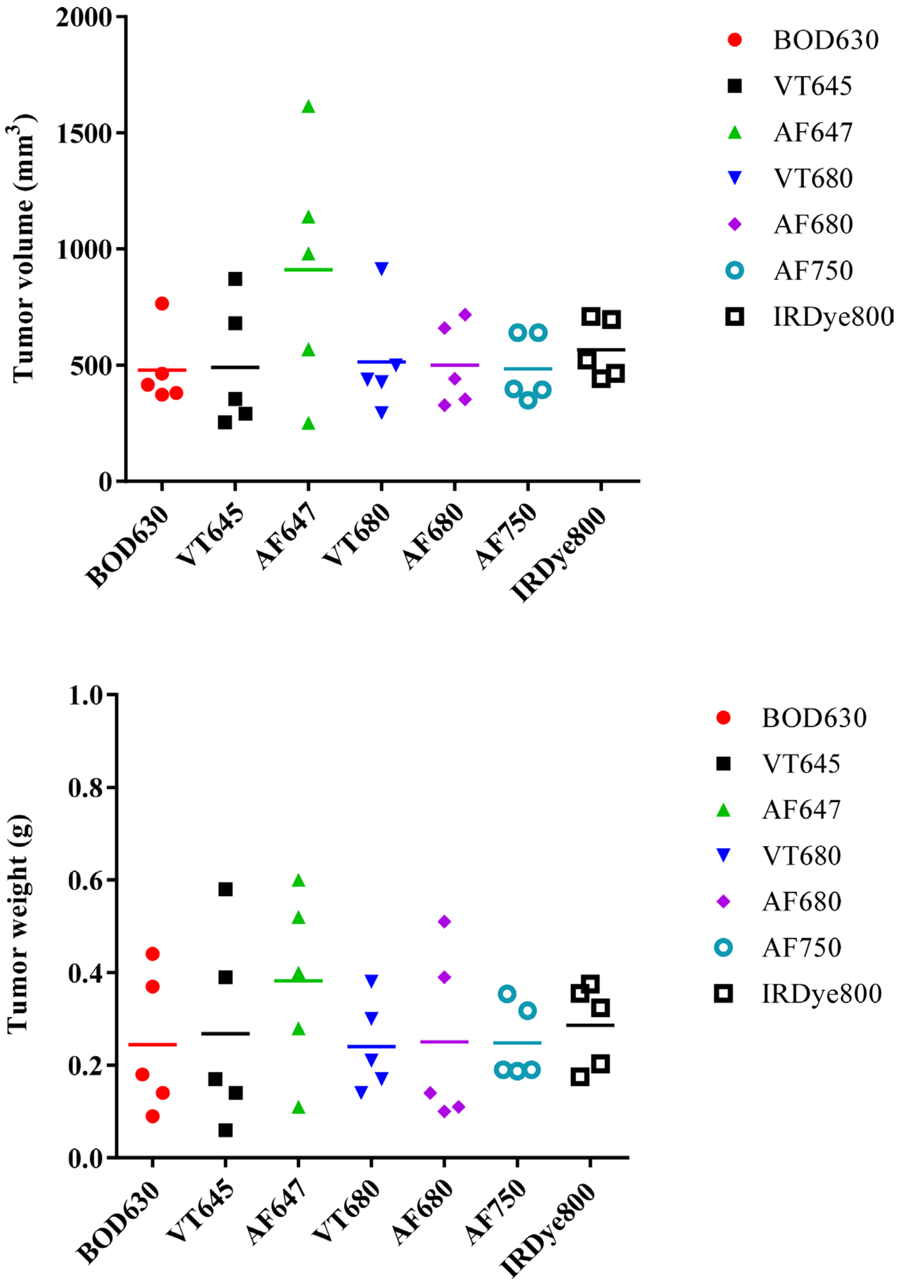

Supplementary Figure 2: Tumor volume (**A**) and Weights (**B**) of various groups at 96 h post-injection.

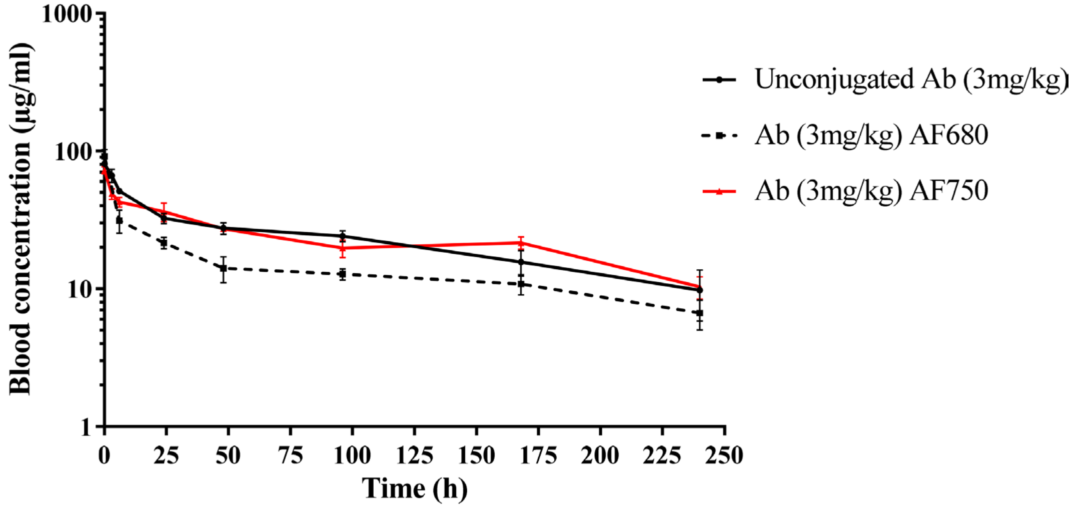

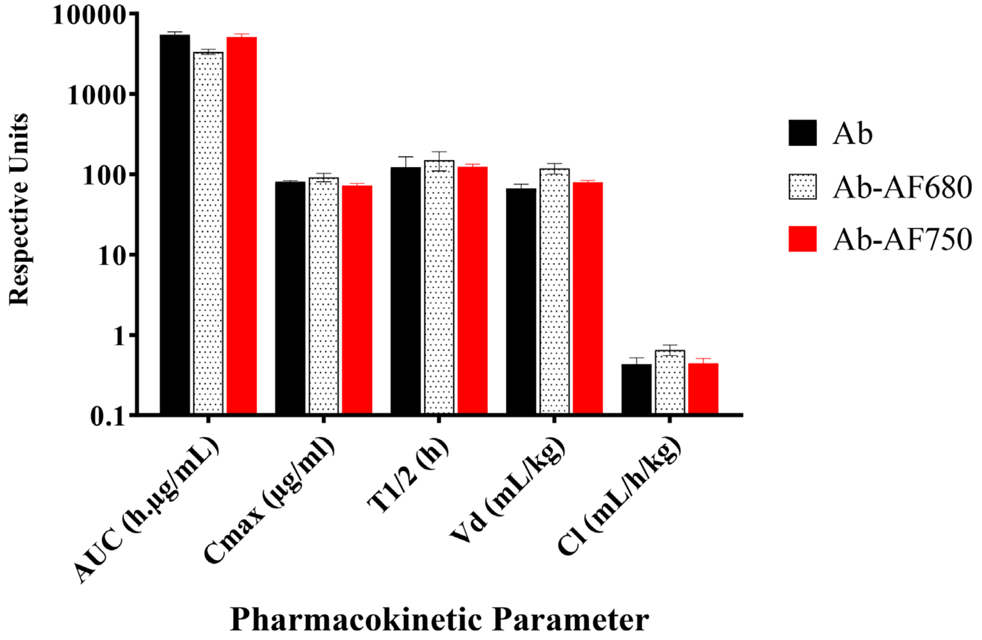

Supplementary Figure 3: Comparison of blood concentration time-profile and PK parameters for unlabeled-Ab, Ab-AF680 and Ab-750. Ab concentrations were measured by LBA assay. PK parameters were similar between unlabeled-Ab, Ab-AF680 and Ab-750.

Supplementary Table 1: Ab Retention in different organs

Fluorophore	% ID/g							
	Tumor	Liver	Spleen	Lung	Kidney	Eyes	Heart	Brain
**BOD630**	5 ± 1	6 ± 0.5	11 ± 6	3 ± 0.5	3 ± 0.4	2 ± 0.3	1 ± 0.3	0.1 ± 0.0
**VT645**	31 ± 3	3 ± 0.4	5 ± 1	10 ± 2	5 ± 1	16 ± 8	4 ± 1	2 ± 0.4
**AF647**	23 ± 3	3 ± 0.2	4 ± 1	8 ± 1	31 ± 0.5	9 ± 2	2 ± 0.4	2 ± 1
**VT680**	15 ± 3	22 ± 2	21 ± 2	7 ± 2	3 ± 1	3 ± 1	4 ± 2	2 ± 1
**AF680**	12 ± 2	1 ± 1	2 ± 2	3 ± 1	0.4 ± 0.2	1 ± 1	1 ± 1	0.1 ± 0.0
**AF750**	24 ± 5	0	0	12 ± 4	2 ± 1	10 ± 3	3 ± 1	1 ± 0.5
**IRDye800**	19 ± 5	7 ± 1	15 ± 4	30 ± 6	5 ± 1	11 ± 3	12 ± 5	5 ± 2

Ab concentration in organs at 96 h for each Ab-F, as calculated from *ex vivo* FMT data. The torso signal from FMT at 5 min and fresh organ weight were used as total dose for the calculation of % ID/g.
